# A pan-cancer analysis of ring finger protein 135 and its relationship to triple-negative breast cancer proliferation and metastasis

**DOI:** 10.18632/aging.204429

**Published:** 2022-12-10

**Authors:** Yiqun Yao, Guanyu Gong, Zijian Guo, Dianlong Zhang

**Affiliations:** 1 Department of Breast and Thyroid Surgery, The Affiliated Zhongshan Hospital of Dalian University, Dalian, Liaoning 116001, China; 2 Department of Oncology, The Affiliated Zhongshan Hospital of Dalian University, Dalian, Liaoning 116001, China

**Keywords:** RNF135, pan-cancer, triple-negative breast cancer, clinical prognosis

## Abstract

Ring finger protein 135 (RNF135) is an E3 ubiquitin ligase with RING finger domains that plays a crucial role in the development of several forms of cancer. Neither the expression profile of RNF135 nor its importance in the diagnosis of pan-cancer have been elucidated as of yet. With the aid of The Cancer Genome Atlas and Gene Expression Omnibus, we have fully mapped the expression profiles, prognostic relevance, genetic modification, immune cell infiltration, and tumor heterogeneity of RNF135 in 33 malignant tumors. RNF135 was expressed inconsistently in various cancers, and variations in RNF135 expression predicted survival outcomes for cancer patients. There was a strong correlation between the levels of the RNF135 genetic mutation and some tumor progression. In addition, a strong correlation was seen between RNF135 expression and immune cell infiltration, tumor mutation burden, microsatellite instability, and immunoregulators. In contrast, the correlation between RNF135 expression and triple-negative breast cancer was investigated in this study. RNF135 may boost the proliferation, migration, and invasion of TNBC cells, according to cell experiments. RNF135 might be utilized as a biomarker to anticipate how a tumor will behave and may have a significant role in how TNBC cells grow and migrate, according to the findings of this study.

## INTRODUCTION

Ring finger proteins are E3 ubiquitin ligases that include the RING finger domain that regulate gene transcription, translation, cell adhesion, and epithelial development [1]. Ring Finger Protein 135 (RNF135) has an N-terminal RING finger domain in addition to C-terminal SPRY and PRY motifs [2]. RNF135 ubiquitinates RIG-I (retinoic acid-inducible gene-I) and enhances its signal transduction potential to create antiviral interferon [2–5]. Additionally, the link between RNF135 and cancer has been shown. RNF135 was related with an increased incidence of malignancy in neurofibromatosis type 1 microdeletion patients [6]. RNF135 is a biomarker and mediator in many human cancers. RNF135’s role in carcinogenesis is unclear.

Cancer treatment has hit a brick wall despite the fact that multiple oncogenes and tumor suppressor genes have been discovered and linked to signaling pathways that regulate cell growth or death in order to generate anti-tumor medications and therapeutic methods [7]. Immunotherapy is seen as a path out of the impasse since it has the potential to result in a paradigm shift in the management of a variety of advanced cancers. Combination therapies made up of immune checkpoint blockade (ICB), radiation, chemotherapy, and metabolic inhibitors are being researched as potential cancer treatments. A novel hypothesis proposes that the tumor microenvironment (TME) is critical for the development and progression of human malignancies [7–9]. It is unclear how these techniques should be used given the complexity of the TME [10]. The tumor microenvironment (TME), one of the primary regulators of immunotherapy effectiveness, may be impacted by oncogene-driven changes in tumor cell metabolism, limiting immune responses and posing challenges for cancer treatment. More research is required to comprehend the dynamic regulatory mechanisms of the matrix and immune components in TME. To confirm new immune-related therapeutic targets for cancer, it is crucial to establish the immunophenotypes of tumor-immune interactions.

Several studies have investigated RNF135 in cancer, including tongue cancer [11], glioblastoma [12, 13] and Hepatocellular Carcinoma [14]. However, the relationship between RNF135 and breast cancer has not been elucidated. Breast cancer is the most prevalent malignant tumor in women, with triple-negative breast cancer (TNBC) having the greatest death rate and the highest likelihood of visceral metastases [15, 16]. Regulation of ubiquitination plays an important role in the occurrence and development of tumors. RNF135, as one of the E3 linked enzymes in the ubiquitination protease system, can bind to a variety of proteins and play a positive regulatory role [17]. RNF135 may have a significant role in the development, progression, and dissemination of TNBC.

This study investigated expression profiles, prognostic relevance, genetic modification, immune cell infiltration, and tumor heterogeneity of RNF135 in pan-cancer. This research confirmed the influence of the RNF135 gene on the *in vitro* migration, proliferation, and cell cycle of MDA-MB-231 and MDA-MB-231-luc cells. Our findings give new insight on the role of RNF135 in the genesis of TNBC and imply that RNF135 might be a therapeutic target for TNBC.

## MATERIALS AND METHODS

### Acquisition and processing of data

We obtained a pan-cancer dataset that was consistently standardized by downloading it from the database maintained by the University of California, Santa Cruz (UCSC) (https://xenabrowser.net/): After obtaining the expression data of the ENSG00000181481 (RNF135) gene from each sample, we used The Cancer Genome Atlas (TCGA), TARGET, and Genotype-Tissue Expression (GTEx) (pan-cancer, *N* = 19131, G = 60499) (Supplementary Table 1). In addition, the sample sources were analyzed using the following criteria: Further log2 (x + 0.001) transformation was applied to each expression value. In the end, cancer species with less than three samples were eliminated, and the results of the expression analysis were obtained for 34 different cancer species.

In addition, we have access to TCGA’s pan-cancer clinical data database in order to improve survival outcomes. In addition, samples with fewer than 30 days of follow-up were removed, and each expression value was transformed using log2 (x + 0.001) both of these processes were taken to guarantee accurate findings. In addition, we excluded cancer species with fewer than 10 samples within a single cancer species, resulting in the expression data of 44 cancer species.

### Differential analysis and prognostic analysis

Unpaired Wilcoxon rank sum and signed rank tests were utilized for significant examination of the differences. Differential expression was determined by comparing the expression levels of normal samples and tumor samples within each tumor. Establishing the Cox proportional hazards regression model required the use of the Coxph function, which was included in version 3.2–7 of the survival software package for R. With this model, which looked at the relationship between gene expression and prognosis in each tumor, the logrank test was used to figure out the difference in prognosis.

### Analysis of the relationship between RNF135 expression and clinical factors

Moreover, we collected information on the expression of the ENSG00000181481 (RNF135) gene in each sample. Each tumor sample’s expression value underwent an additional log2 (x + 0.001) transformation before being analyzed. We excluded cancer species for which there were less than three samples, leaving us with expression data for 26 cancer species. When analyzing the changes in gene expression across samples of tumors at different clinical stages, we made use of the R programming language (version 3.6.4). An unpaired Student’s *t*-test was used to determine the significance of differences between pairs, while an analysis of variance (ANOVA) was employed to determine the significance of differences between several sample groups.

### Immune cell infiltration enrichment

In addition, we retrieved the ENSG00000181481 (RNF135) gene as well as sixty genes representing two distinct kinds of immune checkpoint pathways. We also filtered out all normal samples and then did a log2 (X + 0.001) transformation on each expression value. After that, we determined the Pearson correlation that existed between ENSG00000181481 (RNF135) and the five genes that serve as markers of the immunological pathway. Additionally, we extracted the gene expression profiles of each tumor individually, mapped the expression to GeneSymbol, and made further use of the R packages IOBR (version 0.99.9, https://www.ncbi.nlm.nih.gov/pmc/articles/PMC8283787/) of the Timer method.

### RNF135 SNV and CNV profile in pan-cancer based on GSCA

We obtained the expression data of the RNF135 gene in each sample by downloading a standardized pan-cancer dataset from the UCSC database. The copy number data and the gene expression data of the samples were integrated in the Y Number Variation data set. Then, for each expression value, a log2 (X + 0.001) transformation was applied. At the conclusion of the study, we deleted tumors that had less than three samples from a single species of cancer and gathered expression data for 24 different types of cancer.

We also downloaded software from GDC utilizing MuTect2 (https://doi.org/10.1038/nature08822) in order to handle simple nucleotide level 4 TCGA samples. In the variation data set, we merged the mutation data with the gene expression data of the samples, filtered the samples to remove any synonymous mutations, and then transformed each expression value using a log2 (X + 0.001) transformation. In the end, we took out of consideration cancer samples that had less than three specimens belonging to a single cancer species and obtained expression data for four different types of cancer.

We used R software (version 3.6.4) to calculate the expression difference of genes in clinical stage samples from each tumor, and unpaired Wilcoxon Rank Sum and Signed Rank Tests to determine the significance of differences between pairs. Multiple groups of samples were compared using the kruskal.test statistic.

### RNF135 and tumor heterogeneity in pan-cancer

Using the TMB function of the R software package MafTools (version 2.8.05) Burden, we computed the tumor mutation burden (TMB) for each tumor. In the end, we chose to eliminate cancer species that had fewer than three samples for each kind of cancer that we studied and obtained expression data for 37 cancer types [18, 19].

For each tumor instability score, we merged the microsatellite instability (MSI) and gene expression data of the samples. We then transformed each expression value using log2 (X + 0.001) to account for the different cancer types. In the end, we chose to eliminate cancer types that had fewer than three samples for each kind of cancer and instead gathered expression data for 37 types of cancer [20, 21].

### Culture and transfection of TNBC cells

Both MDA-MB-231 and MDA-MB-231-luc cells were obtained via separate purchases made at the American Type Culture Collection in VA, USA, and Cell Biolabs in CA, USA. At a temperature of 37 degrees Celsius and a relative humidity of 5% CO2, the Leibovitz L-15 medium was used to cultivate the cells, with the addition of 10% fetal bovine serum and 1% antibiotic-antimycotin. The aforementioned findings were from research conducted by Life Technologies (NY, USA). Cells were cultured in a gel mixture that included 6 mg/ml matrigel (Corning, Lot# 5061003), 0.5 mg/ml collagen (Corning, Lot# 5092001), 3.7 g/L NaHCO3, and 0.05 M HEPES. This was done so that 3D cultures could be created.

Utilizing the pLVTHM-GFP lentiviral RNAi expression system, lentiviruses expressing human RNF135 short hairpin RNA were produced (TOP: GATCCGCAGGCCCTGTCTTCTGGAAAGCATTTTCAAGAGAAATGCTTTCCAGAAGACAGGGCCTGTTTTTTC; BOTTOM: AATTGAAAAAACAGGCCCTGTCTTCTGGAAAGCATTTCTCTTGAAAATGCTTTCCAGAAGACAGGGCCTGCG). In order to infect the cells, lentiviral particles bearing either specific or negative vectors were used. Flow cytometry using FACS was used to examine the polyclonal cells that were chosen based on the presence of GFP signals. Using quantitative real-time PCR, it was possible to prove that RNF135 had a high level of success when it came to transfection.

### Cell cycle analysis via flow cytometry

After harvesting the cells, they were planted on 6-well plates. Following an incubation period of 24 hours, the cells were collected, digested, and then centrifuged at a temperature of 4°C for 5 minutes at 1000 revolutions per minute. After rinsing with ice-cold PBS, the deposited cells were resuspended in 70% ethanol at 4°C for 30 minutes. The cells were then rinsed and resuspended in 100 mL of PBS containing 50 mg/mL of RNase A (Sigma, MO, USA) and 0.25 % Triton X-100 at 4°C for 30 minutes. This procedure was performed to eliminate any leftover RNase A. Finally, the cells were stained for 30 minutes with 10 mg/mL of propidium iodide (PI; lot: 1685935, Life Technologies, NY, USA) at a concentration of 1685935. Then, a FACScan flow cytometer was used to conduct an immediate study of the cells (Becton Dickinson, CA, USA). After each round of testing, the proportion of cells in each phase of the cell cycle was counted for each sample.

### Quantitative real-time PCR

RNA was extracted from MDA-MB-231 cells using the RNA isoPlus^®^ Reagent Kit (Catalog Number: RR820A, Takara Biotechnology), and the process was carried out in accordance with the technique that had been described before. In order to convert RNA into cDNA, a kit called PrimeScript^®^ RT Reagent Kit (Catalog Number: RR037A, Manufacturer: Takara, Shiga, Japan) was used. The cDNA was amplified by running it through the 7500 Real-Time PCR System while using the SYBR^®^ Premix Ex Taq^™^ Kit (Applied Biosystems, 7500 Real Time PCR System, Thermo, USA). The following describes the criteria for the cycling: 40 cycles at temperatures of 60°C for 34 seconds, 95°C for 5 seconds, and 95°C for 30 seconds. For the analysis, the comparative Ct technique was used, and GAPDH served as the loading control for the genes of interest. The primers were as follows: RNF135 (Forward: 5′-TACTGGGAAGTGGACACTAGGAATT-3′, reverse: 5′-CTTGACCATGTGCCATGCA-3′); GAPDH (Forward: 5′-GCACCGTCAAGGCTGAGA AC-3′, reverse: 5′-TGGTGAAGACGCCAGTGGA-3′).

### Migration assay

In order to evaluate the capacity of cell lines to migrate, a wound healing experiment was carried out. Cells at a concentration of 1 × 10^4^/ml were cultivated on a 6-well plate at 37°C with 5% CO_2_ for 24 hours, and the cells were allowed to develop until they reached 80% confluence. Was added to the culture for a period of 24 hours. A sterile, disposable serological pipette with a capacity of 20 L was used to make a scratch in the form of a straight line on the cells. After being cultivated in DMEM without FBS for twenty-four hours, the cells were washed with one milliliter (mL) of DMEM to remove any debris and to smooth the edge of the scratch. A microscope was used in order to capture pictures of the multiplication of the cells. Image J was used for the calculation, which determined the proportion of the migratory area (1.48v, National Institute of Health, USA).

### Cell proliferation assay

Cells were seeded using 96-well plates at a density of 2 × 10^5^ cells per well. Following a series of incubations at 37 degrees Celsius and 5% carbon dioxide for varying amounts of time, the CCK-8 kit that was supplied by Tiangen (Hangzhou, China) was mixed at a concentration of 10 microliters per well, and the cells were then incubated for three hours at 37°C and 5% carbon dioxide. In the last step, the absorbance at 450 nm (Thermo Fisher Scientific, Inc.) was measured with a microplate reader.

### Animals

Ten female severely combined immunodeficiency (SCID) mice weighing 28–32 g and aged 5 weeks were obtained from the Experimental Animal Center of Dalian Medical University (Dalian, China). The animals were kept with a 12-hour light/dark cycle and ad libitum access to food and drink. All experimental methods were conducted in compliance with the National Institutes of Health’s standards and recommendations. The Experimental Animal Committee at Dalian Medical University authorized this work (Dalian, China).

### Experimental metastasis breast cancer models

Each mouse was injected intravenously (tail vein) with MDA-MB-231-Luc cells (106 in 0.1 ml PBS) expressing shRNA targeting RNF135 (shRNF135) or control shRNA (sh-) to facilitate the spread of breast cancer cells. After 5 weeks, tumor metastasis was tracked by intraperitoneal administration of 150 mg/kg D-luciferin, and a bioluminescence imaging of animals was done using conventional display procedures using the IVIS^®^ Spectrum system (PerkinElmer, Waltham, MA, USA).

### Statistical analysis

In order to evaluate the disparities between the groups, ANOVA was carried out. In order to do statistical analysis, the SPSS 25.0 program was used. Each experiment was carried out three times by its own dedicated researcher. When *P* was less than 0.05, statistical significance was assumed.

## RESULTS

### RNF135 expression in pan-cancer

**Figure 1:**
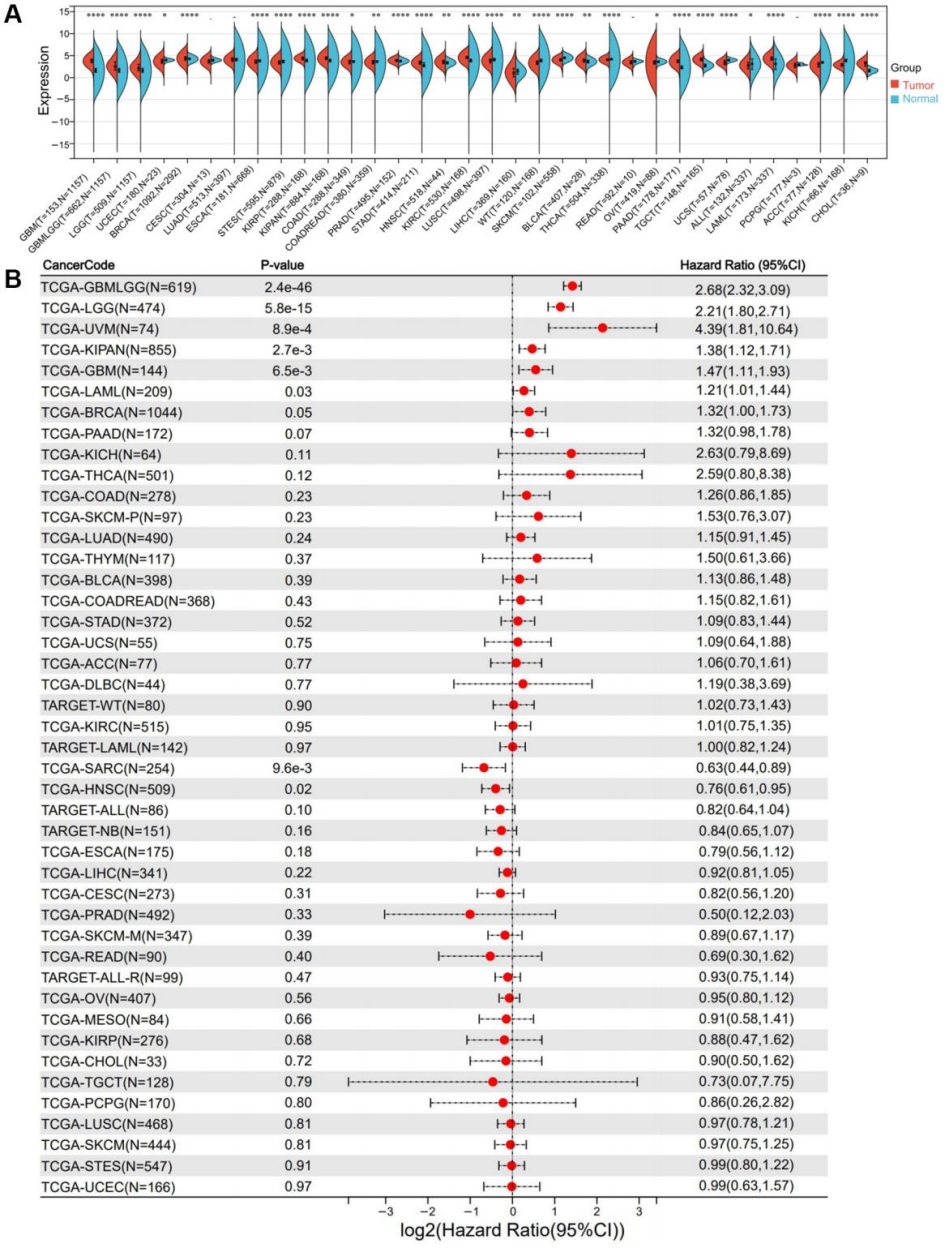
**Differential expression and prognostic survival analysis of RNF135.** (**A**) Expression of RNF135 mRNA in pan-cancer. (**B**) Prognostic survival of RNF135 mRNA in pan-cancer.

Using RNA-seq data from 33 different kinds of TCGA malignancies, an investigation was conducted on the mRNA expression of RNF135 in pancreatic cancer patients. Upregulation was reported to a substantial degree in 15 cancers such as glioblastoma multiforme (*P* = 8.1e-79), glioma (LGG) (*P* = 1.2e-71), brain lower grade glioma (GBMLGG) (*P* = 3.5e-34), breast invasive carcinoma (BRCA) (*P* = 1.1e-5), et al. Significant downregulation was detected in 15 tumors, such as uterine corpus endometrial carcinoma (*P* = 0.04), esophageal carcinoma (*P* = 3.3e-5), stomach and esophageal carcinoma (*P* = 3.9e-11), stomach and esophageal carcinoma (*P* = 0.03), et al. (Figure 1A). RNF135 is differentially expressed in the majority of malignancies, but its expression pattern varies among tumors.

### The relationship between RNF135 and pan-cancer survival

This study observed that seven tumor types, such as GBMLGG (*P* = 2.4e-46, HR = 2.68), LGG (*P* = 5.8e-15, HR = 2.21), BRCA (*P* = 0.05, HR = 1.32), the pan-kidney cohort (KIPAN) (*P* = 2.7e-3, HR = 1.38), GBM (*P* = 6.5e-3, HR = 1.47), Uveal Melanoma (*P* = 8.9e-4, HR = 4.39), and Acute Myeloid Leukemia (*P* = 0.03, HR = 1.21) increased with poor prognosis (Figure 1B). Low expression is associated with poor prognosis in two tumor types (sarcoma (*P* = 9.6e-3, HR = 0.63) and head and neck squamous cell carcinoma (HNSC) (*P* = 0.02, HR = 0.76) (Figure 1B). Furthermore, we discovered that low RNF135 expression was associated with poorer overall survival (OS) (*P* = 1.3e-08) and disease-free survival (DFS) (*P* = 8.5e-14) in the total cancer population (Supplementary Figure 1). It is difficult to generalize what impact the expression of RNF135 will have on the prognosis of various malignancies. A poorer prognosis is associated with a high level of RNF135 expression in BRCA because its level of expression is higher in BRCA tissues than in normal tissues. There is a possibility that RNF135 is a significant oncogene in BRCA.

### RNF135 expression correlated with clinical feature of pan-cancer

**Figure 2:**
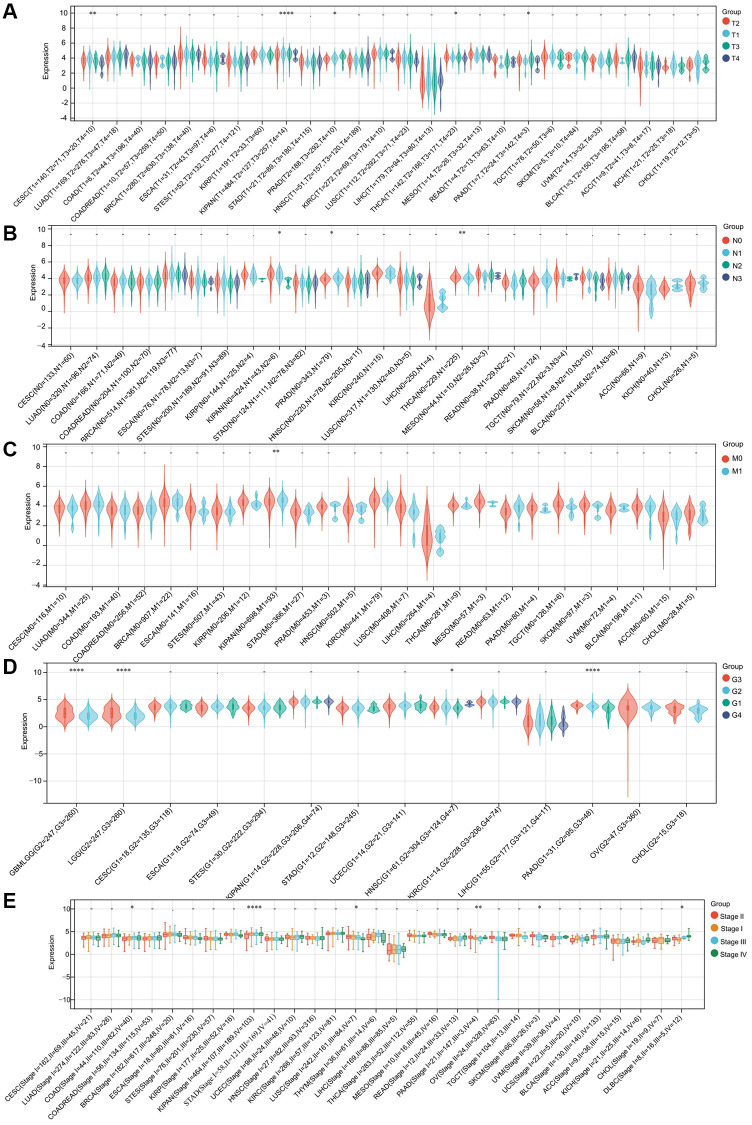
**The correlation between RNF135 expression and clinical characteristics of pan-cancer.** (**A**) The relationship between RNF135 expression and T stage. (**B**) The relationship between RNF135 expression and N stage. (**C**) The relationship between RNF135 expression and M stage. (**D**) The relationship between RNF135 expression and Grade. (**E**) The relationship between RNF135 expression and clinical stage. ^*^P < 0.05, ^**^P < 0.01, ^***^P < 0.001.

In addition, we started observing the expression of RNF135 in various clinical phases of pan-cancer. There were statistically significant differences found in the T-stage of five different cancers, including cervical squamous cell carcinoma and endocervical adenocarcinoma (CESC) (*P* = 6.6e-03), Kipan (*P* = 8.2e-05), prostate adenocarcinoma (PRAD) (*P* = 0.02), thyroid carcinoma (THCA) (*P* = 0.02), and pancreatic adenocarcinoma (PA) (Figure 2A). In the N-stage of three cancers, such as KIPAN (*P* = 0.03), PRAD (*P* = 0.01), and THCA (*P* = 3.2e-03) (Figure 2B). We noticed a statistically significant change in the M-stage of one tumor, Kipan (*P* = 5.8e-06) (Figure 2C). The grades of four cancers differed significantly: GBMLGG (*P* = 4.6e-09), LGG (*P* = 4.6e-09), HNSC (*P* = 0.02), and PAAD (*P* = 4.3e-08) (Figure 2D). Six tumor stages exhibited statistically significant changes, including colon adenocarcinoma (*P* = 0.05), KIPAN (*P* = 7.6e-07), lung squamous cell carcinoma (*P* = 0.01), PAAD (*P* = 4.1e-03), skin cutaneous melanoma (*P* = 0.04), and lymphoid neoplasm diffuse large B-cell lymphoma (*P* = 0.02) (Figure 2E). RNF135 may be intimately associated with the development and progression of pancreatic cancer.

### RNF135 expression and immune infiltrating level correlation in pan-cancer

**Figure 3:**
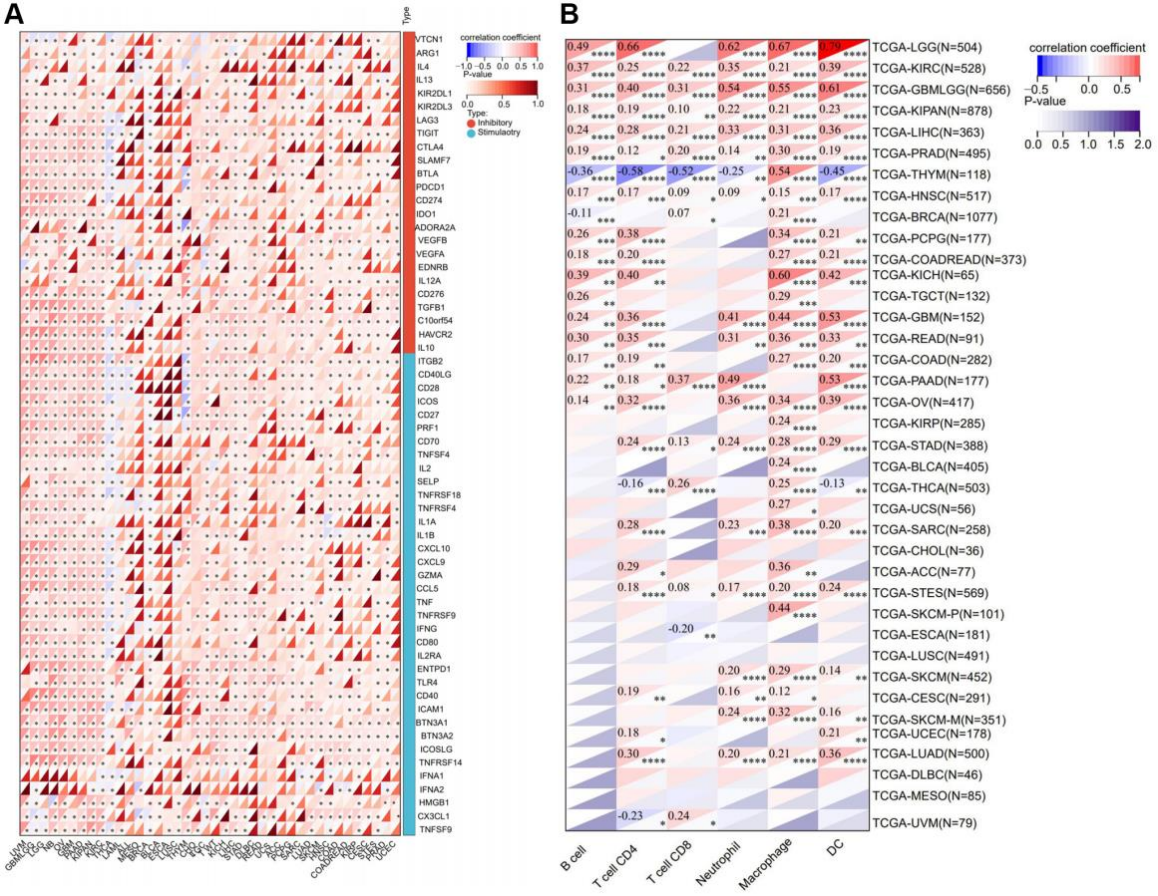
**Correlation analysis between the expression of RNF135 and immune microenvironment.** (**A**) Correlation analysis between the expression of RNF135 and immune checkpoint inhibitors. (**B**) Correlation analysis between RNF135 and immune cell infiltration in pan-cancer. ^*^P < 0.05, ^**^P < 0.01, ^***^P < 0.001.

Pan-cancer investigations aimed at elucidating the immunological function of RNF135 are essential for identifying the sorts of malignancies that may respond favorably to anti-RNF135 immunotherapy. Our data demonstrated a favorable correlation between RNF135 and the majority of immunomodulators in pan-cancer (Figure 3A). Finally, Timer scores were derived for 9406 tumor samples representing 38 distinct tumor categories. We found a substantial link between RNF135 gene expression and immune infiltration in 34 different cancer species by using the coefficient to build the highly related immune infiltration score (Figure 3B). There is a strong link between the expression of RNF135 and a number of immune checkpoint genes and the invasion of different immune cells in BRCA.

### The correlation between gene mutation and RNF135 mRNA

**Figure 4:**
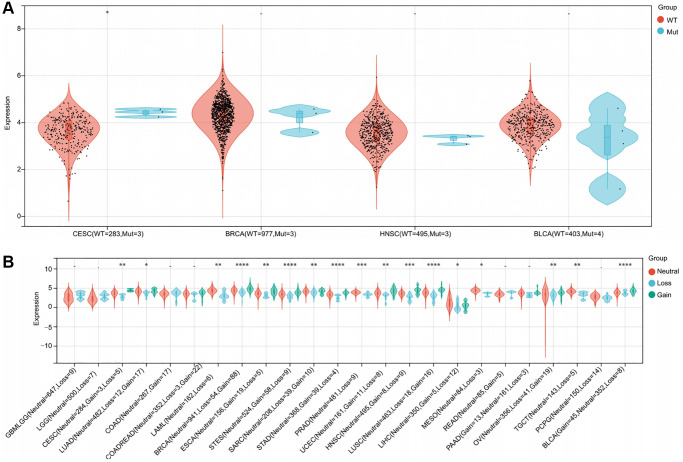
**An illustration of the relationship between RNF135 expression and gene mutation.** (**A**) A Spearman association between RNF135 SNV and mRNA was performed in pan-cancer. (**B**) A Spearman association between RNF135 CNV and mRNA was performed in pan-cancer. ^*^P < 0.05, ^**^P < 0.01, ^***^P < 0.001.

A Spearman relationship was performed between RNF135 single nucleotide variants (SNV) and mRNA in pan-cancer. We noticed a statistically significant difference in 1 tumor, CESC (*P* = 0.04) (Figure 4A). CESC (*P* = 1.3e-03), lung adenocarcinoma (*P* = 0.02), acute myeloid leukemia (*P* = 2.7e-03), BRCA (*P* = 8.6e-17), esophageal carcinoma (*P* = 4.8e-03), and stomach and esophageal carcinoma (*P* = 1.2e-07) exhibited significant differences in RNF135 copy number variations (CNV) and mRNA (Figure 4B).

### RNF135 is linked to the TMB and MSI in some cancers

**Figure 5:**
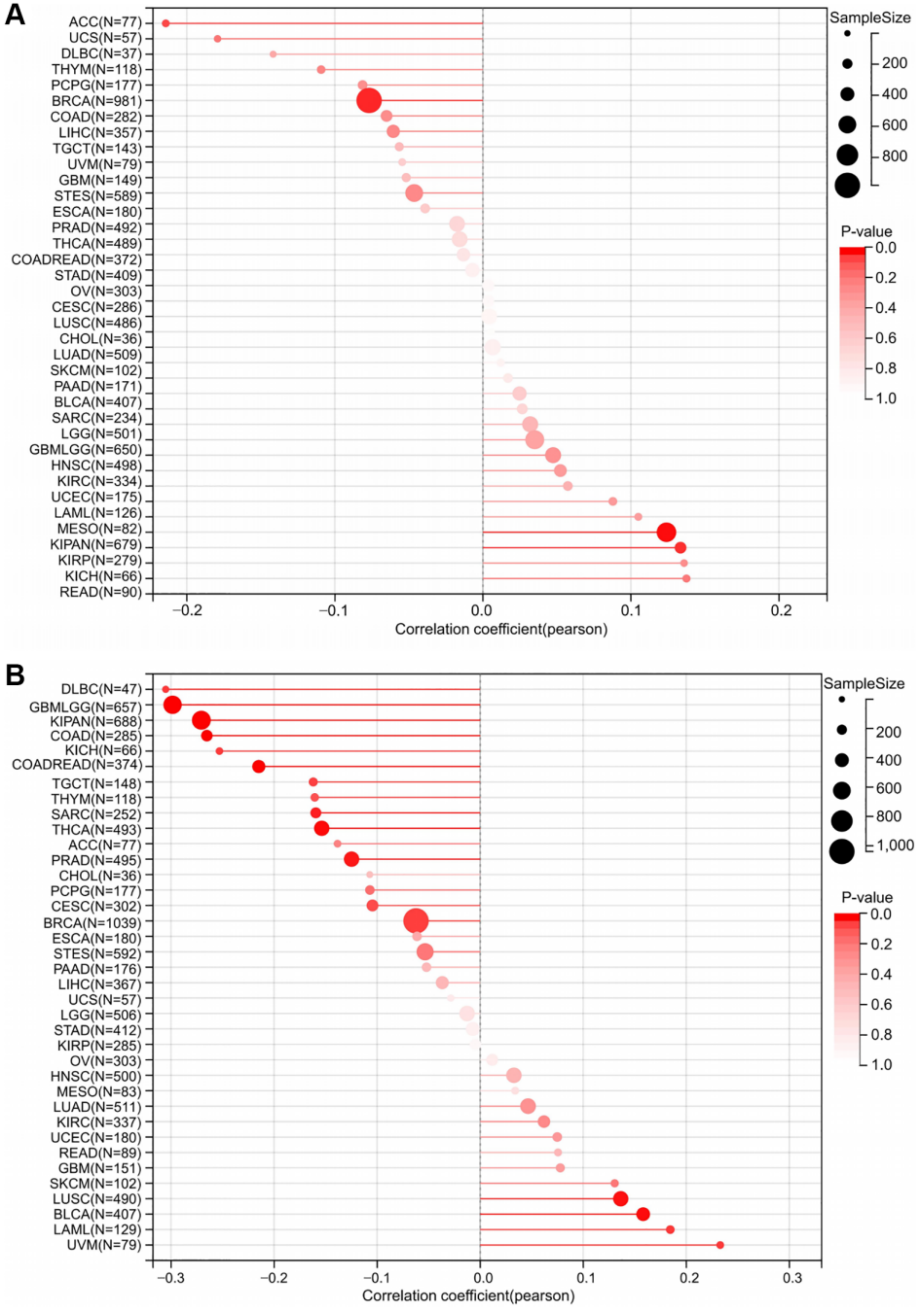
**A strong connection between RNF135 expression and tumor heterogeneity.** (**A**) Correlation between RNF135 expression and Tumor mutation burden. (**B**) Correlation analysis of RNF135 expression and Microsatellite instability.

We investigated their Pearson correlation in each tumor and found a significant association in TMB in three cancers, with a strong positive correlation in two tumors, such as KIRP (*P* = 0.025) and KIPAN (*P* = 0.001) in one kind of tumor (Figure 5A). We evaluated their Pearson correlation in each tumor and found a significant relationship between MSI and 15 malignancies, with a significant positive correlation in 4 tumors and a significant negative correlation in 11 tumors (Figure 5B). TMB and MSI have a substantial association with BRCA, which is not difficult to determine. This suggests that RNF135 is intimately associated with the tumor heterogeneity of BRCA and has significant implications for BRCA.

### The effects of RNF135 on MDA-MB-231 cell proliferation, migration, and invasion

**Figure 6:**
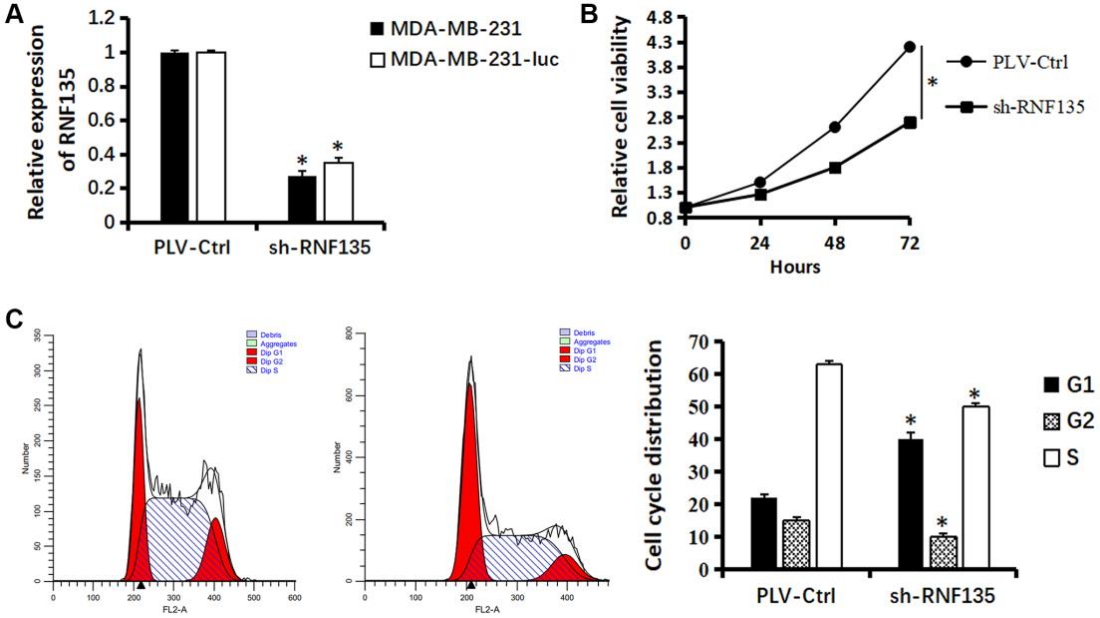
**Relationship between RNF135 and viability of MDA-MB-231 cells.** (**A**) qRT-PCR assay shows the transcriptional levels of the RNF135 gene with GAPDH used as the loading control in MDA-MB-231 and MDA-MB-231-luc cells. (**B**) Effect of sh-RNF135 on the proliferation of MDA-MB-231 cells was detected by CCK-8 assays. (**C**) The cell cycle distribution of MDA-MB-231 cells were measured using propidium iodide staining and flow cytometry analyses. Data are presented as the mean ± SD for three independent experiments (^*^P < 0.05) (Original magnification: 100×).

**Figure 7:**
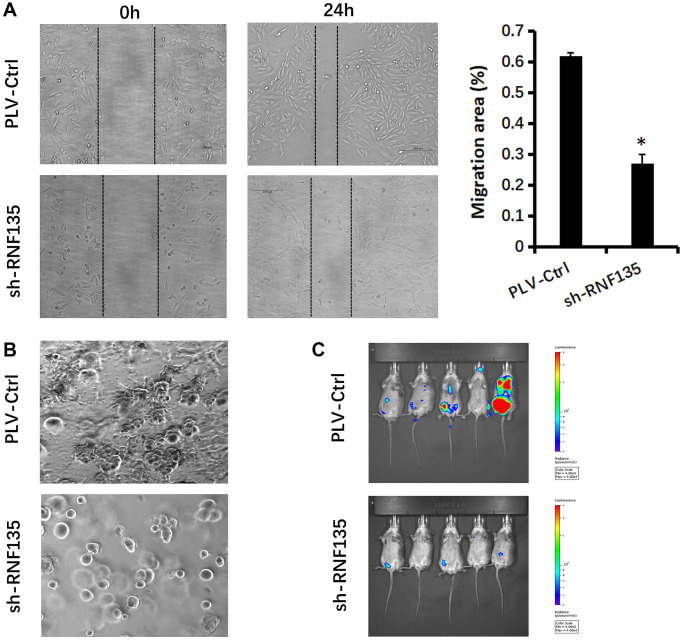
**Relationship between RNF135 and migration ability of MDA-MB-231 cells.** (**A**) Migration of MDA-MB-231 cells was detected via wound healing assay. (**B**) Changs in cellular morphology of MDA-MB-231 cells were investigated via 3D culture. (**C**) Bioluminescent imaging of mice harboring metastases after tail vein injection of MDA-MB-231-luc cells at week 5. Data are presented as the mean ± SD for three independent experiments (^*^P < 0.05) (Original magnification: 100×).

In order to evaluate the impact of RNF135, lentiviral shRNA vectors were used to suppress RNF135 expression in MDA-MB-231 and MDA-MB-231-luc cells in a stable and specific way. Expression levels were measured using quantitative real-time PCR (Figure 6A). CCK8 results showed that RNF135 down-regulation significantly suppressed the cell viability of MDA-MB-231 cells (Figure 6B). Flow cytometry data showing 39.86% G0/G1 phase cells in the shRNF135 cells and 22.28% G0/G1 phase cells in the PLV-Ctr cells suggest that RNF135 knockdown arrested the cell cycle in the G0/G1 phase (Figure 6C). Wound healing results showed that shRNF135 significantly decreased the migration capacity of MDA-MB-231 cells (Figure 7A). Reduced RNF135 expression in 3D cultures hindered MDA-MB-231 cell invasiveness (Figure 7B). Next, MDA-MB-231-luc cells were injected into the tail veins of SCID mice to examine the possible role of RNF135 in breast cancer metastasis (Figure 7C). We found that RNF135 knockdown significantly lowers the number of metastases compared to vector control.

## DISCUSSION

E3 ubiquitin-protein isopeptide ligases were identified as potential biomarkers and therapeutic targets in a wide range of cancers [22]. Research has shown that the gene known as RNF135, which was discovered in the course of the genome project, is responsible for neurofibromatosis as well as other hereditary conditions that affect humans. Researchers think that a change in the expression of RNF135 may have a role in the onset of disease [23].

Several ring finger proteins are protein ubiquitin ligases. This action is important in many biological processes [24]. Previous research has shown that RNF149 inhibits the increase in cell proliferation caused by BRAF wild type [1]. It has been proven that RNF43 is overexpressed in colorectal cancer and stimulates cancer cell proliferation [25]. It’s a new tumor-associated antigen and cancer immunotherapy target [26]. RNF135 may bind multiple proteins due to the presence of a ring finger and a PY motif, demonstrating its broad range of actions, including its association with EGFR [27]. Many studies have looked at how RNF135 affects the growth and migration of human glioblastoma. Reduced expression of RNF135 by elemene inhibits human glioblastoma proliferation and migration [12]. Targeting RNF135 and downregulating miR-485-3p enhanced glioblastoma cell proliferation and migration [13]. Earlier research established that RNF135 influences the growth of human glioblastoma through the ERK pathway [14]. Moreover, it was proven that ring finger proteins may have a key role in the development of cancer. To investigate the biological role of RNF135, we reduced RNF135 expression in TNBC cell lines using shRNA. Next, we conducted a number of tests and discovered that significantly lessening RNF135 expression with shRNA prevented TNBC cell lines from proliferating and migrating *in vitro*.

The immune system is able to recognize and eliminate tumor cells from the TME. Nevertheless, tumor cells may avoid the immune system via a variety of survival and development mechanisms. TIICs alter the prognosis of individuals with various cancers [28]. We compared the expression of 40 immunological checkpoint genes to RNF135. TIICs that express PD-1 and PD-L1 have a poorer prognosis and histopathological grade [29]. RNF135 expression correlated with tumor purity but not TIICs. Overexpression of RNF 135 was linked to bad outcomes. RNF135 expression was linked to tumor invasion. MSI enhanced the incidence of cancer with particular clinicopathological characteristics, including TMB and lymphocyte infiltration. TMB was a latent ICB biomarker [30]. TMB may predict immune-related survival in breast cancer patients [31]. In the future, we will be able to measure the success of immunotherapy by assessing RNF135 expression, and we will be able to mix a targeted RNF135 treatment with standard immunotherapy to boost its efficacy.

Despite our research and assimilation of data from several sources, the current study had substantial limitations. To verify our findings and expand their therapeutic value, more *in vitro* or *in vivo* studies are necessary. Our understanding of RNF135’s role in cancer was greatly aided by bioinformatics, and molecular biology confirmed that it promoted cancer in TNBC. However, more *in vitro* or *in vivo* studies are required to confirm our findings and maximize their therapeutic potential. It was unclear if RNF135 altered clinical survival via immunity, despite its relationship with immunity and clinical survival in human malignancies. Our results demonstrated the significance of RNF135 in tumor immunology, metabolic activity, and epithelial-to-mesenchymal transition, as well as a mechanism by which it influences these processes. Future research on RNF135 expression and the immunological environment of the tumor may aid in the development of immunotherapy-based cancer therapeutics.

RNF135 is aberrantly elevated in several tumor tissues, and its aberrant expression is associated with the prognosis of malignancies. The aberrant expression of RNF135 is associated with immune cell infiltration, the expression of immunological checkpoints, and tumor heterogeneity in pan-cancer. Additionally, knockdown of RNF135 may influence TNBC cell growth and transformation. Therefore, RNF135 is a promising biomarker for prognosis.

## Supplementary Materials

Supplementary Figure 1

Supplementary Table 1

## Abbreviations

RNF135: Ring finger proteins 135
TNBC: Triple-Negative Breast Cancer
RIG-I: retinoic acid-inducible gene-I
ICB: immune checkpoint blockade
TME: tumor microenvironment
UCSC: University of California Santa Cruz
TCGA: The Cancer Genome Atlas
GTEx: Genotype-Tissue Expression
TMB: Tumor mutation
MSI: Microsatellite instability
SCID: severely combined immunodeficiency
LGG: Glioma
GBMLGG: Brain Lower Grade Glioma
BRCA: Breast invasive carcinoma
KIPAN: Pan-kidney cohort
OS: overall survival
DFS: disease free survival
CESC: Cervical squamous cell carcinoma and endocervical adenocarcinoma
PRAD: Prostate adenocarcinoma
THCA: Thyroid carcinoma
PAAD: Pancreatic adenocarcinoma
HNSC: Head and Neck squamous cell carcinoma
SNV: single nucleotide variants
CNV: copy number variations
TIICs: Tumor infiltrating immune cells
