# Supplementary material for: A pan-cancer analysis of ring finger protein 135 and its relationship to triple-negative breast cancer proliferation and metastasis
Source: Aging (Albany NY). 2022 Dec 10;14(23):9758–72. doi: 10.18632/aging.204429 (PMC9792201; doi:10.18632/aging.204429)
Supplement: Supplementary Figure 1 [file aging-14-204429-s001.pdf]

## SUPPLEMENTARY FIGURE

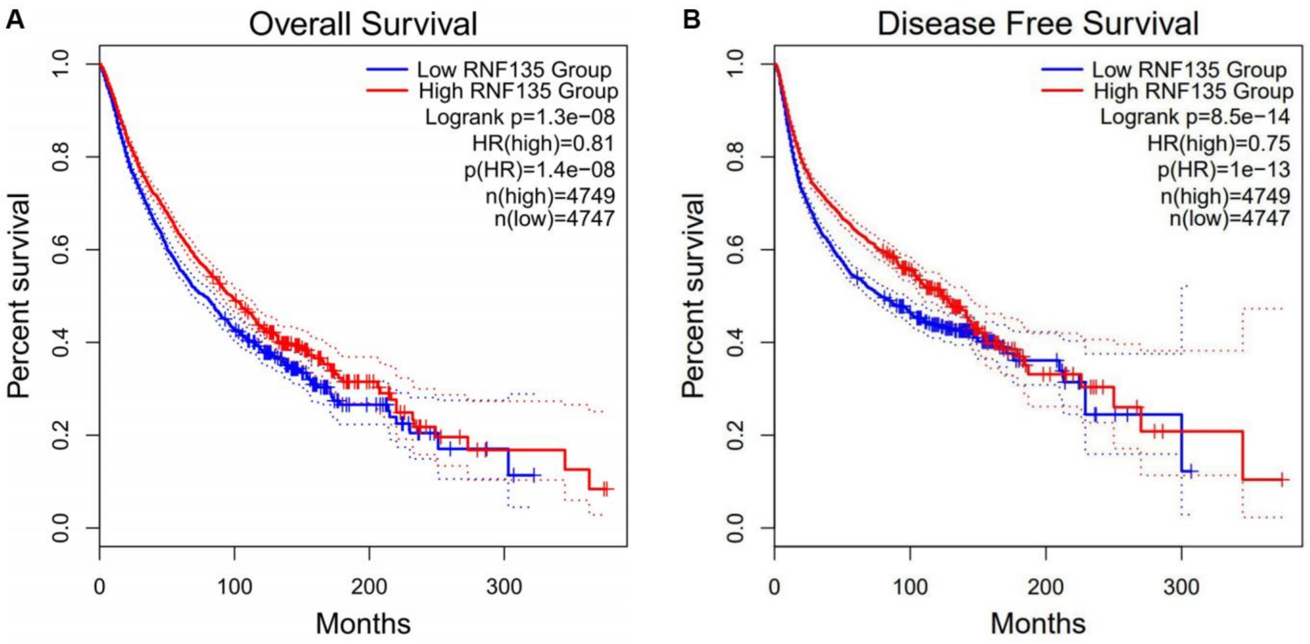


**Supplementary Figure 1. Relationship between RNF135 expression and prognosis of pan-cancer patients.** (**A**) Association between RNF135 expression and overall survival in cancer patients. (**B**) Association between RNF135 expression and disease free survival in cancer patients.
